# Recurrent ovarian torsion challenges in management and saving fertility: A rare case report

**DOI:** 10.1016/j.ijscr.2025.112039

**Published:** 2025-10-10

**Authors:** Hadeel Halabe, Maya Shahoud, Yaman Houari, Mahmoud Qasab, Dalaa Shahoud, Ashraf Olabi

**Affiliations:** a University of Aleppo, Faculty of Medicine, Aleppo, Syria

**Keywords:** Ovarian torsion, Mature cystic teratoma, Dermoid cyst, Gynecologic emergency, Ovarian preservation

## Abstract

**Introduction:**

Ovarian torsion is a time-sensitive gynecologic emergency that may lead to irreversible ovarian damage. The condition becomes particularly critical in women with a solitary ovary, where timely diagnosis is essential to preserve fertility.

**Case presentation:**

A woman of reproductive age with a history of right oophorectomy due to torsion of a mature cystic teratoma presented to the emergency department with acute lower abdominal pain, nausea, and vomiting. Pelvic ultrasound revealed the absence of the right ovary and a complex left ovarian cyst measuring 6 × 6 cm, with signs of congestion. Exploratory laparotomy revealed a twisted, violaceous left ovary measuring approximately 10 × 10 cm with two full twists of the infundibulopelvic (IP) and utero-ovarian (OU) ligaments. Detorsion was performed.

**Discussion:**

Current evidence challenges traditional practices of routine oophorectomy in torsion, as histologic studies report preserved viability in 43 % of ovaries despite ischemic appearance. This case reinforces the feasibility of detorsion in reproductive-aged women, particularly those with a solitary ovary, to safeguard fertility. The absence of contralateral ovarian tissue further underscores the need for conservative surgical approaches.

**Conclusion:**

This report emphasizes the critical role of early recognition and conservative surgical management in ovarian torsion, especially in patients with a solitary ovary. Timely detorsion can prevent irreversible fertility loss, even in grossly ischemic-appearing ovaries. Patient education about acute pelvic pain and clinician vigilance in high-risk populations are essential to mitigate diagnostic delays.

## Highlights

• A rare case of ovarian torsion in a young woman with previous oophorectomy highlights the risk of recurrence and treat to fertility.
• Acute abdominal pain with prior adnexectomy raised diagnostic challenges, especially in a setting without access to laparoscopy.
• Conservative surgery preserved ovarian function, confirmed by hormonal levels, menstrual return, and ultrasound findings.
• This case underscores the importance of early diagnosis and fertility-sparing management in reproductive-age women with adnexal torsion.

## 1 Introduction

Teratomas are germ cell tumors that contain well-differentiated tissues. Ovarian teratomas are broadly classified histologically into monodermal teratomas, immature teratomas, and mature cystic teratomas. The most common type among these tumors is the mature cystic teratoma (MCT), also known as a dermoid cyst [1]. In the majority of cases, MCTs occur in women of reproductive age and are usually unilateral, while only about 10 % of cases are bilateral [2]. In rare cases, MCT may rupture, which can lead to acute abdominal pain and surgical emergency. In contrast, adnexal torsion with MCT is common. Ovarian torsion is one of the most common gynecologic surgical emergencies. It is a relatively rare condition, accounting for about 2.7 % of surgical emergencies, and ranks as the fifth most common gynecological surgical emergency [3]. Ovarian torsion occurs when the ovary twists over the ligaments that support it in the adnexa. The fallopian tube often twists with the ovary and is then referred to as adnexal torsion. Early diagnosis of ovarian torsion is crucial to fertility preservation, especially in young women. Unfortunately, late diagnosis can result in ovarian tissue necrosis [4]. In this case, we report a rare case of ovarian torsion with MCT in a young woman with history(12 years ago)of right ovariectomy due to torsion of contralateral ovary and we highlight the challenges encountered in managing this unique case as per the SCARE checklist [5].

## 2 Case presentation

**Fig. 1:**
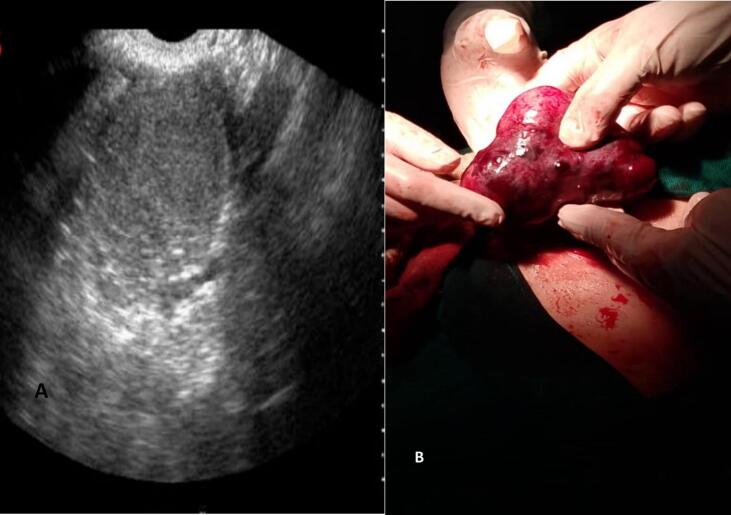
A “In this image, The left ovary is enlarged, has an abnormal gray scale appearance and appears heterogeneous.”. B The left ovary appears enlarged, congested, firm, violaceous in color, and twisted twice around its pedicle.

A 23 -year -old woman (G2P2) came to the emergency department of our hospital complaining of acute abdominal pain. The onset of symptoms was three hours ago. The pain was sharp and radiated to the left iliac fossa. The patient reported nausea and vomiting without diarrhea. Her temperature and blood pressure were normal, pulse 110/min. Medical history was clear. Gynecological history: right adnexectomy for torsion of MCT 12 years ago. Gynecological examination revealed tenderness and rebound tenderness in the left iliac fossa. Transabdominal ultrasound revealed an enlarged left ovary with a heterogeneous hemorrhagic cyst roughly 6 cm posterior to uterus. The right ovary was absent. Endometrial thickness was 5.7 mm (Fig. 1A).

The next planned step was laparoscopy; however, due to its unavailability in our facility, the decision was made to proceed with a diagnostic laparotomy (Table 1).

**Table 1:** The following laboratory tests were performed pre-operatively HCG; human chorionic gonadotropin, WBC; white blood cell, RBC; red blood cell INR; international normalized ratio APTT; activated partial thromboplastin time.

Laboratory investigation	Laboratory value	Normal reference range
Beta hCG	<5 mIU/Ml	Non-pregnant: <5 mIU/mL
		Pregnant: >5 mIU/mL
Blood group	O+	RH positive
Complete blood count		
WBC count	8.8k/μL	3.6–11k/μL
RBC count	4.3	3.80–4.80 MIL/μL
Hemoglobin blood	13.3	12–15 g/Dl
Hematocrit	38.40 %	36 %–46 %
Platelet count	149k/μL	150–410k/μL
Coagulation profile		
Prothrombin time	17.5 s	11.5–14.5 s
INR	1.11	0.80–1.20
APTT	35.2 s	28.6–38.2 s
C-reactive protein	2	Up to 5 mg/L

**Fig. 2:**
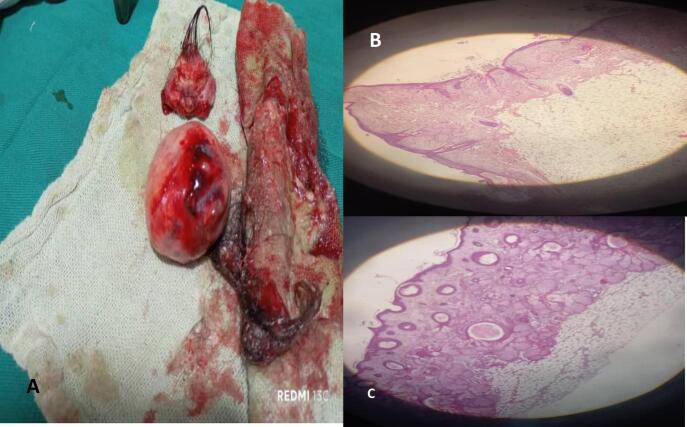
A “In this image, two mature cystic teratomas that were excised from the left ovary are shown.”. B and C Microscopic image confirm the diagnosis of ovary torsion due to dermoid cysts.

**Fig. 3:**
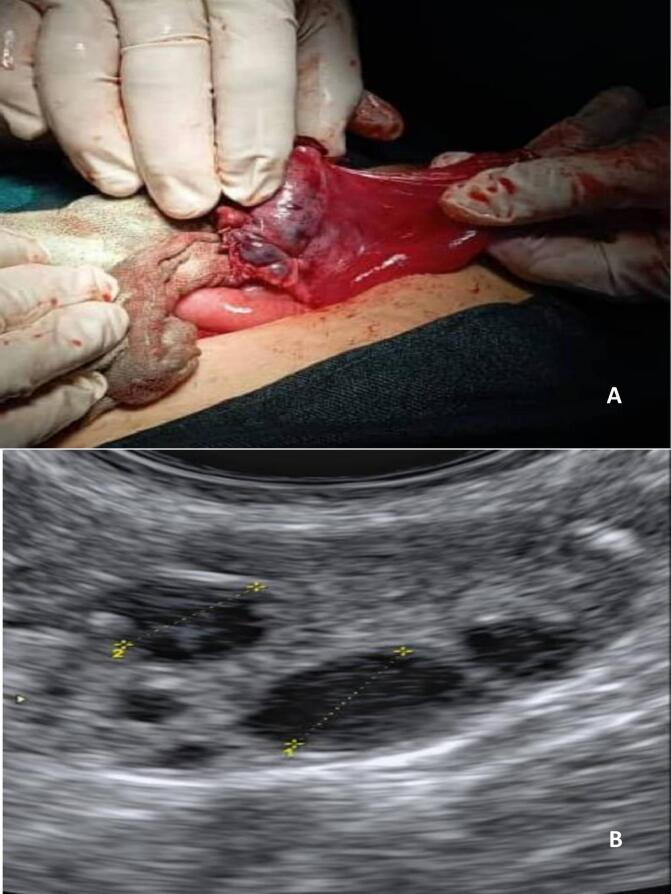
A: Ovarian preservation was achieved after confirming reperfusion and restoration of the ovary's normal reddish coloration. B: On day 13 of the subsequent menstrual cycle, a dominant follicle was identified on vaginal ultrasound diameter 3 cm.

An exploratory laparotomy was performed; uterus appeared normal; right ovary was absent. There was an enlarged left ovary 10 × 10 cm that was congested, firm, violaceous in color, with two full twists of the infundibulopelvic ligament (Fig. 1B). Detorsion was carefully performed, followed by the enucleation of two dermoid cysts (Fig. 2A). Lab results and histology studies supported our findings (Fig. 2B, C). Ovarian preservation was achieved after confirming reperfusion and restoration of the ovary's normal reddish coloration (Fig. 3A).

Postoperatively, the patient had an uneventful recovery. Pelvic ultrasound one week after surgery revealed no abnormalities or adnexal lesions. Two weeks later, the patient experienced a spontaneous menstrual period, indicating successful preservation of ovarian function. After two months, serum levels of follicle-stimulating hormone (FSH) and anti-Müllerian hormone (AMH) were measured and found to be within normal reference ranges (Table 2).

**Table 2:** The following laboratory tests were performed postoperative.

Laboratory investigation	Laboratory value	Normal reference range
Anti-Müllerian hormone (AMH)	65.3 ng/ml	1.20_9.05 ng/ml
Follicle-stimulating hormone	8 mIU/ml	Follicular phase: 3.5–12.4 mIU/ml Ovulation phase: 4.7–21.5 mlU/mL Luteal phase: 1.7–7.7 mIU/mL Post-menopause: 25.8–135 mIU/ml

Transvaginal ultrasonography revealed an ovary of normal dimensions, with few antral follicles. Follow-up was conducted. On day 13 of the subsequent menstrual cycle, a dominant follicle was identified on ultrasound (Fig. 3B).

## 3 Discussion

Ovarian torsion is a complete or partial rotation of an ovary along the supporting ligaments that can lead to partial or complete ovarian blood supply obstruction [4]. It contributes to 2.7 % of all gynecological emergencies [3] and frequently leads to ovary removal. MCT commonly occurs in women of reproductive age [3]. MCT (Mature Cystic Teratoma) is a tumor composed of differentiated somatic cells from germ cells in the ectoderm, endoderm, and mesoderm. Its benign or malignant nature depends on tissue maturity. Ovarian teratomas can be bilateral in 10 % of cases. The mean age at diagnosis is 38 years [2]. Ovary torsion bilateral in 1 % of cases [6]. Risk factors for torsion include the presence of an ovarian mass or cyst, particularly those larger than 5 cm, as well as a history of pelvic surgery, especially previous torsion and tubal ligation. Ovarian torsion may present with variable symptoms [7]. Usually, patients present with acute abdominal pain along with nausea, vomiting, and tachycardia [4]. Ovarian torsion is a diagnostic challenge particularly in reproductive age. Ectopic pregnancy must always be ruled out first with a serum beta hCG test. A negative beta hCG essentially excludes pregnancy. Ruptured ovarian cysts and ovarian torsion can have similar presentations and may both show free fluid in the pelvis on ultrasound. However, cyst rupture typically presents with a sudden onset of sharp pain, often occurring during or after sexual intercourse. Tubo-ovarian abscesses usually present with gradually worsening lower pelvic pain accompanied by fever. Appendicitis can also present with right lower quadrant pain, nausea, vomiting, and fever; laboratory findings may include leukocytosis [8]. Ultrasound (transabdominal or transvaginal) is the first-line imaging modality for suspected ovarian torsion. Ultrasound cannot rule out ovarian torsion as the sensitivity ranges from 35 to 85 %. Pelvic examination has poor sensitivity in detecting an ovarian mass or cyst, and the reliability of pelvic examinations are further reduced by obesity and age > 55. To make a definitive diagnosis of ovarian torsion is for a surgeon to intraoperatively visualize the twisted ovary [9]. Recently, conservative surgical treatment of adnexal torsion was suggested as the primary treatment process in order to preserve long-term fertility, particularly in young women of reproductive age. This approach corresponds to an increasing amount of data supporting the avoidance of oophorectomy whenever possible [10]. This case represents a classic example of preserving ovarian function as during the second menstruation after surgery, the patient's clinical and laboratory evaluations suggested continued ovarian function with normal level of follicle-stimulating hormone (FSH) and anti-Müllerian hormone (AMH). Furthermore, the patient was asymptomatic, without symptoms of surgical menopause [11]. Microscopic pathology studies of ovarian tissue samples from patients who underwent oophorectomy for ovarian torsion showed that 43 % did not have evidence of ischemia. This observation highlights the possibility that a significant number of oophorectomies may have been unnecessary, since the ovary was still functional. These findings support the conservative surgical management of patients with adnexal torsion to improve chances of future fertility [12].

## 4 Conclusion

Early diagnosis of ovarian torsion is crucial to prevent serious complications that can arise from delayed treatment. It is essential to educate patients about the importance of seeking immediate medical attention in the event of acute abdominal pain. Prompt intervention can significantly impact outcomes and preserve ovarian function. It is prudent to avoid surgical removal when possible as this can lead to catastrophic consequences for the patient's health and fertility. Raising awareness and encouraging timely responses can make a significant difference in patient outcomes.

## CRediT authorship contribution statement

The work's conception and design: all authors.
Paper writing, and article revision: all authors.
Final revision and approval: all authors.

## Informed consent

Unnecessary, information taken from the patient's file.

## Consent for publication

All authors provide consent for publication.

## Ethical approval

This case report does not require ethical approval as it involves a single patient case that is anonymized and does not include any identifiable personal information. The patient provided informed consent for the publication of this report.

## Guarantor

Ashraf Olabi.

## Research registration number

Our research study does not involve human subjects.

## Provenance and peer review

Not commissioned, externally peer-reviewed.

## Funding

There are no funding sources.

## Declaration of competing interest

The authors declare that they have no competing interests.
